# Evidence of climate‐driven regime shifts in the Aegean Sea’s demersal resources: A study spanning six decades

**DOI:** 10.1002/ece3.8330

**Published:** 2021-11-13

**Authors:** Dimitrios Damalas, Vasiliki Sgardeli, Paraskevas Vasilakopoulos, Georgios Tserpes, Christos Maravelias

**Affiliations:** ^1^ Hellenic Centre for Marine Research Institute of Marine Biological Resources and Inland Waters Heraklion Greece; ^2^ Department of Ichthyology and Aquatic Environment University of Thessaly Volos Greece; ^3^ Joint Research Centre European Commission Ispra Italy

**Keywords:** climate change, Integrated Ecosystem Assessment, Integrated Resilience Assessment, Mediterranean Sea, multivariate analysis

## Abstract

Climate change (CC) can alter the configuration of marine ecosystems; however, ecosystem response and resilience to change are usually case‐specific. The effect of CC on the demersal resources of the Aegean Sea (east Mediterranean Sea) was investigated during the past six decades applying a combination of multivariate analysis, non‐additive modeling and the Integrated Resilience Assessment (IRA) framework. We focused on the study of: (i) the biological “system” complex, using proxies of biomass (landings per unit of capacity) for 12 demersal taxa, and (ii) the environmental “stressor” complex, described by 12 abiotic variables. Pronounced changes have occurred in both the environmental and biological system over the studied period. The majority of the environmental stressors exhibited strikingly increasing trends (temperature, salinity, primary production indices) with values started exceeding the global historical means during late 1980s‐early 1990s. It is suggested that the biological system exhibited a discontinuous response to CC, with two apparently climate‐induced regime shifts occurring in the past 25 years. There is evidence for two‐fold bifurcations and four tipping points in the system, forming a folded stability landscape with three basins of attraction. The shape of the stability landscape for the Aegean Sea's biological system suggests that while the initial state (1966–1991) was rather resilient to CC, absorbing two environmental step‐changes, this was not the case for the two subsequent ones (intermediate: 1992–2002; recent: 2003–2016). Given the current trajectory of environmental change, it is highly unlikely that the biological system will ever return to its pre‐1990s state, as it is entering areas of unprecedented climatic conditions and there is some evidence that the system may be even shifting toward a new state. Our approach and findings may be relevant to other marine areas of the Mediterranean and beyond, undergoing climate‐driven regime shifts, and can assist to their adaptive management.

## Graphical abstract

Applying a combination of multivariate analysis, non‐additive modelling and the Integrated Resilience Assessment (IRA) framework, we provide evidence that after the 1990s, the Aegean Sea has most likely entered areas of unprecedented climatic conditions, altering the biological system and making it highly unlikely to return to its pre‐1990s state.

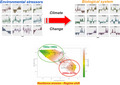

## 1 INTRODUCTION

Over 90% of the energy gained by the Earth as a result of human activities accumulates in the ocean, and most of it stays in the upper 700 m of the water column (Rhein et al., 2013). Hence, the oceans “feel” most of the impacts of climate change (CC). The impacts are not geographically homogeneous, with the North Atlantic and Mediterranean Sea storing a disproportionally large stock of anthropogenic carbon compared to other ocean regions (Khatiwala et al., 2013). Climate variability and change affect marine species in a multitude of ways from eliciting physiological responses and changing productivity and survival, to altering habitat and resource availability that lead to range expansions/contractions and shifts of geographical distributions (Koenigstein et al., 2016).

Apart from direct effects, a change of abiotic conditions may affect species indirectly through their interactions with other species. Due to the interconnectedness of biological systems, CC may trigger a complete reorganization of an ecosystem, known as “regime shift” (Scheffer, 2009; Scheffer & Carpenter, 2003). Regime shifts are described as abrupt changes of the ecosystem state (e.g., characterized by different dominance patterns) and can be triggered even by smooth, continuous climatic shifts, particularly in ecosystems with low ecological resilience, for example, as a result of anthropogenic pressures (fishing, pollution, habitat change). Typically, after a regime shift, an ecosystem state is self‐stabilizing through positive feedback loops and the restoration of the environment to previous conditions does not necessarily cause a recovery of the ecosystem to its previous state (hysteresis) (Scheffer, 2009). In this context, alternate regimes refer to dynamic states that can be viewed as basins of attraction around the system response curves (attractors), forming a folded stability landscape on the system‐stressor surface (Barnosky et al., 2012; Scheffer, 2009)—see Figure S0. Systems within a folded stability landscape may switch to a new state either through a “horizontal movement” across a tipping point associated with a change in stressors, or through a “vertical movement” associated with a small change in the system state while the system lies close to a tipping point (Scheffer, 2009).

Two major climatic shifts have been documented in the North hemisphere during the 20th century: in the mid‐1970s and the late 1980s (Möllmann & Diekmann, 2012 and references therein). Both were related to a change of the North Atlantic Oscillation (NAO) Index, which was in turn associated with an increase in sea temperature. Concurrent with these climatic shifts, several north European marine ecosystems (North Atlantic, North and Baltic Seas, Scotian shelf, and Black Sea) were reported to have undergone ecosystem regime shifts (Möllmann & Diekmann, 2012). All these studies suggest that besides fishing, CC is a significant factor shaping the fluctuations of marine resources (Barange et al., 2018).

In the Mediterranean Sea, a regime shift in oceanographic variables and planktonic assemblages has been documented in the western part of the basin in the late 1980s (Conversi et al., 2010). Additionally, regime shifts in fisheries resources have been reported across the Mediterranean basin over the past 30 years (Tzanatos et al., 2014), with such shifts being the result of ecosystem reorganizations as a discontinuous response to sea warming (Vasilakopoulos et al., 2017). Furthermore, the exploitation rate of Mediterranean stocks over the past 20 years has been steadily increasing, selectivity (proportional exploitation of juveniles) has been deteriorating, and stocks have been shrinking (Vasilakopoulos et al., 2014). It is likely that Mediterranean fisheries vulnerability to CC is higher, given overfishing, higher exposure to warming, arrival of non‐indigenous species, and an overall lower adaptive capacity (Hidalgo et al., 2018).

Herein, we combine multivariate and nonadditive modeling tools to identify plausible regime shifts in the demersal community of the Aegean Sea, implementing the Integrated Resilience Assessment (IRA) framework (Vasilakopoulos & Marshall, 2015; Vasilakopoulos et al., 2017). We apply this framework to an empirical biological data‐series (fisheries‐dependent relative biomass) during 1966–2017, and investigate the effect of the concurrent development of a series of environmental variables. The analysis of this exceptionally long time‐series allows us to unveil previously unknown climate‐driven ecosystem dynamics in the Aegean Sea and may assist in future adaptive management of the local marine biological resources.

## 2 MATERIALS AND METHODS

### 2.1 Study area‐fisheries

**FIGURE 1:**
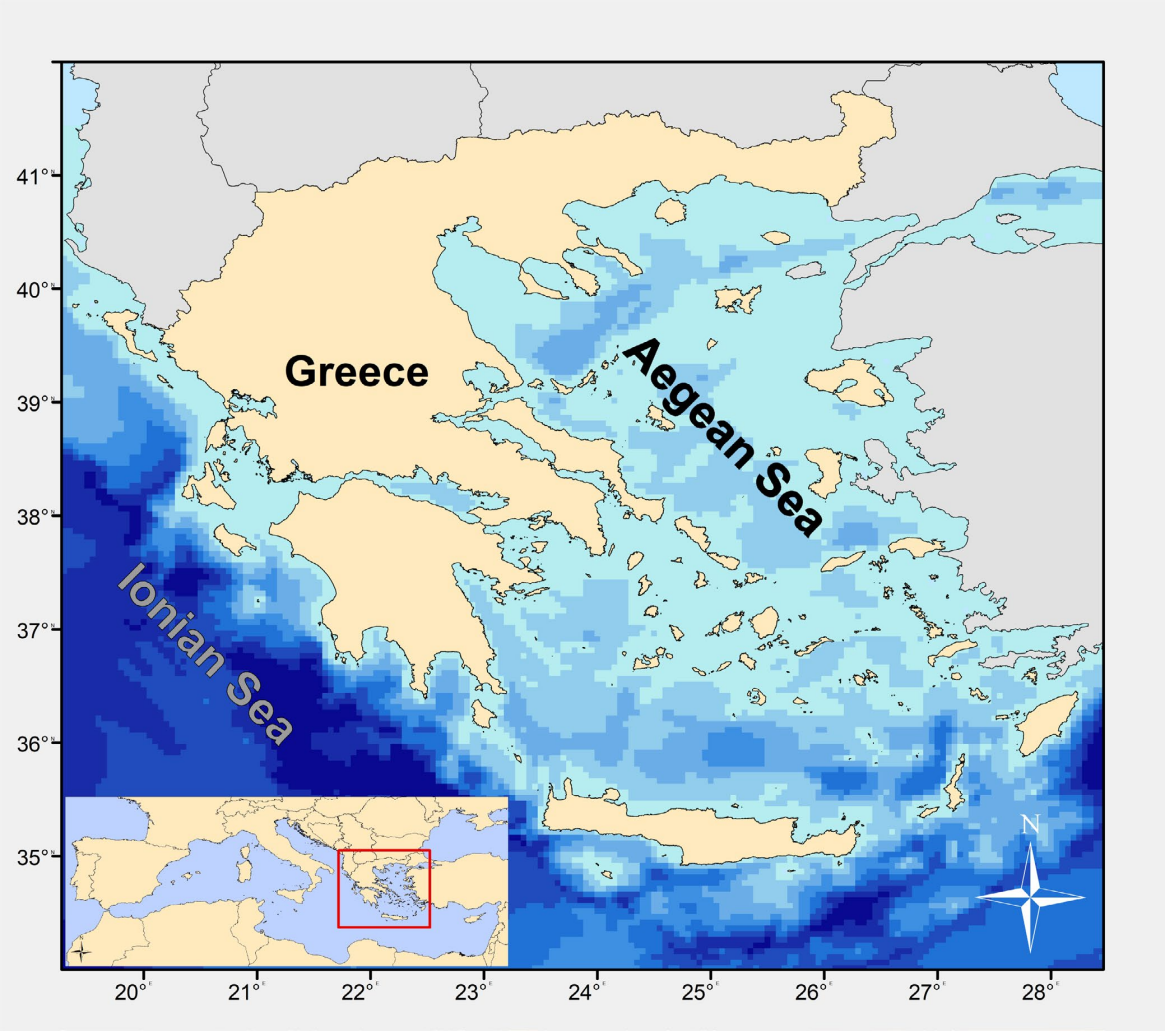
Bathymetric map of the study area

The study area covers the Aegean Sea (Figure 1), located in the Eastern Mediterranean Sea. Generally, the region has a complex geomorphology, which directly or indirectly influences the fishing activity; the Aegean can be divided in two distinctive ecoregions: the south Aegean is an oligotrophic area where evaporation exceeds freshwater income, while the north Aegean is influenced by the cold, brackish, nutrient‐rich Black Sea waters (Lykousis et al., 2002; Zervakis et al., 2004). As a result, more than 50% of the Greek otter bottom trawl fleet targeting demersal species, operates in the north Aegean Sea, producing >57% of the total national demersal landings; north Aegean bottom trawl landings exceed those in the south Aegean by a factor of more than 5 (HELSTAT, 1964–2017). Approximately 15,000 commercial vessels are involved in the Greek demersal fisheries (using primarily bottom trawls, static nets and longlines), 12,000 of which are exerting their effort in the study area (Aegean Sea) (HELSTAT, 1964–2017). The Greek demersal fisheries are multispecies in nature targeting a highly diversified mix of fish, cephalopods, and crustaceans. Management is conducted through input controls (spatio‐temporal effort restrictions) and various technical measures (Kapantagakis, 2007).

### 2.2 Data

#### 2.2.1 Environmental

A large number of historical physicochemical variables for the Aegean Sea during the period 1966–2017 were compiled (Table S1). Data were made available through the Horizon2020 CERES project (http://ceresproject.eu/). They were derived from the POLCOMS‐ERSEM coupled model framework, producing daily, monthly, and annual outputs of physical, chemical, and biological variables at a 1/10 of a degree spatial resolution. POLCOMS (Proudman Oceanographic Laboratory Coastal Ocean Modelling System, Holt et al., 2001) is a 3‐D community physical model with the ability to run in regions that include both the deep ocean and the continental shelf. The European Regional Sea Ecosystem Model (ERSEM; Baretta et al., 1995) is one of the most complex lower trophic‐level marine ecosystem models currently in use, including bacteria, four phytoplankton and three zooplankton functional groups, a fully resolved diurnal cycle, variable carbon to chlorophyll ratios and independent nutrient pools for carbon, nitrogen, phosphorous, and silicate. The POLCOMS‐ERSEM modeling system is well established in the NE Atlantic and has been applied successfully in the Mediterranean (Butenschön et al., 2016; Holt et al., 2001, 2012; Kay & Butenschön, 2016). Model outputs have been validated through comparisons to satellite values. A full description of data specifications can be traced in CERES (2018).

To shortlist the environmental variables of interest to this study, we focused on their relevance for the demersal resources (“system”). From the full set of 36 environmental variables, some refer to the sea bottom (e.g., ben_DOC‐benthic dissolved carbon), others are by definition 2D (e.g., optical depth), while most of them were available at different depth zones (3D variables, e.g., gross primary production). From a series of experimental surveys in the Aegean Sea (Katsanevakis et al., 2009), most species/taxa analyzed herein (see *Demersal resources* below) exhibited higher abundance at depths of <50 m. Hake differentiated from this general pattern, showing high abundance both in waters beyond the continental shelf (Katsanevakis et al., 2009; Labropoulou et al., 2008; Sion et al., 2020) as well as in shallower waters (PANDORA, 2019). Hake abundance is linked to the occurrence of chlorophyll‐a fronts and high densities are to be expected after local phytoplankton blooms (Cartes et al., 2004). Alemany et al. (2014) and Druon et al. (2015) showed that surface oceanic features are strong drivers even for demersal resources, such as hake. Sion et al. (2020) found that Mediterranean hake biomass indices exhibited similar trends for sea bottom temperature and sea surface temperature. Sea surface temperature is typically used to express the preferred temperature of demersal species (e.g., Cheung et al., 2013 and subsequent literature on the Mean Temperature of the Catch—MTC) and to capture the effect of warming upon marine ecosystems, including demersal resources, within Integrated Ecosystem Assessments (IEA; e.g., Möllmann & Diekmann, 2012 and references therein). Moreover, employing correlation analysis on the available variables by depth, confirmed that the concern over the choice of depth can be relaxed, since their values at different depths were highly correlated (Figure S2). Consequently, for 3D variables it was decided to use yearly averages at 5 m depth over the whole Aegean Sea area. To avoid duplicating information, correlation testing (Szekely et al., 2007) was used to remove highly correlated variables, ending up with a subset of 12 variables (Figure S2; Figure S1): T, S, ssh, grossPP, pH, O, ben_DOC, MLD, Chl1, Chl2, Chl3, P3C (full description in Table 1).

**TABLE 1:** List of environmental variables used in the analyses

Variable	Units	Description
T (SST)	°C	Water potential temperature
S	psu	Salinity
ssh	m	Sea surface height
grossPP	tons C/day	Gross primary production
pH	−log[H+]	Degree of acidity/alkalinity
O	mmol m^3^	Dissolved oxygen
ben_DOC	mg C m^2^	Benthic dissolved organic carbon
MLD	m	Mixed layer depth
Chl1	mgCHL m^3^	Diatoms Chl
Chl2	mgCHL m^3^	Nanophytoplankton Chl
Chl3	mgCHL m^3^	Picophytoplankton Chl
P3C	mg C/m^3^	Picophytoplankton C

#### 2.2.2 Demersal resources

Marine fisheries landings and fleet capacity data for the Greek fleet have been recorded and published in yearly bulletins since January 1964 by the Hellenic Statistical Authority (HELSTAT, 1964–2017). Landings refer to the quantity of each main taxon caught, while effort and capacity data, matching the official landings data, refer to fleet size (Nb of boats), engine horsepower (HP or kW), and boat tonnage (GT). More elaborate measures of fishing effort, such as days at sea (DAS), engine power per DAS (kW × DAS), number of hauls, hours of hauling, length of nets or number of hooks deployed, are not available before 2003; the EU Fisheries Data Collection Framework (DCF) was officially launched in Greece in late 2002. The use of HP or kW as a surrogate for effort would have led to unreliable estimates, due to the renowned underdeclaration of engine power (Damalas et al., 2014; DG MARE, 2019); as a result, Gross Tonnage (GT) was used as the most consistent capacity measure of the fleet targeting demersal stocks. GΤ has been successfully applied in an analogous long‐term study in the Mediterranean Sea (Fortibuoni et al., 2017).

Landings of demersal species in the Aegean Sea account for ~25% of total Greek landings (HELSTAT, 1964–2017). In this study, 12 demersal taxa were considered, based on the availability of uninterrupted time series, and were expressed in Landings Per Unit of Capacity (LPUC), defined as the sum of annual landings of each species from the demersal fleets (trawlers and small‐scale fishery) divided by the sum of GT of these fleets (kg/GT). To confirm that this GT‐based LPUC was a good proxy of biomass, we investigated the correlation between LPUC and LPUE (effort in GT x DAS, kW x DAS) for the most recent years (e.g., >2002) that detailed effort data were available through the EU DCF. A statistically significant positive correlation was confirmed (*p* < .05—Figure S3).

**FIGURE 2:**
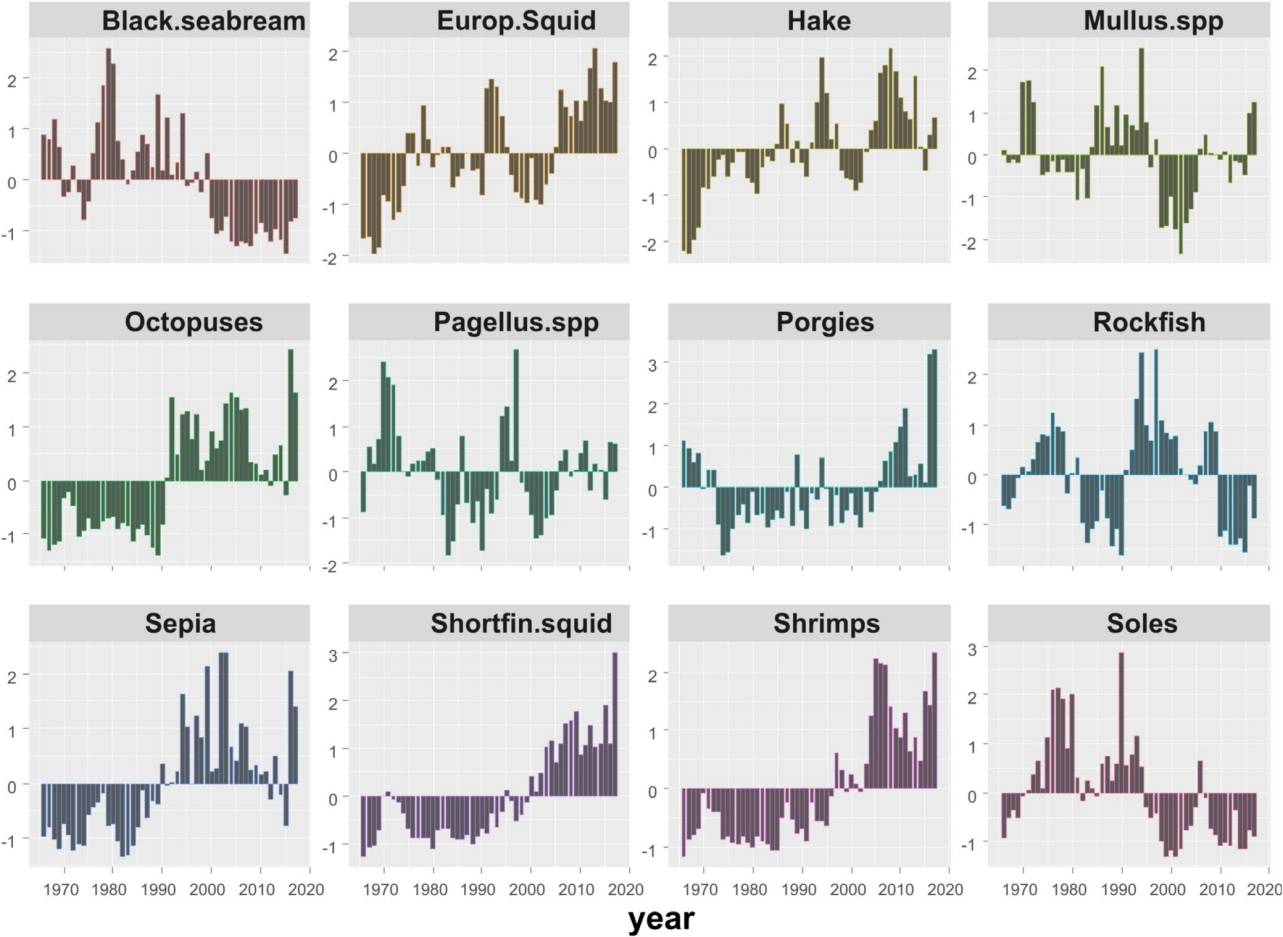
Anomaly of LPUC in kg/GT expressed as difference from the overall mean divided by the standard deviation for 12 demersal species/taxa landed in the Aegean Sea during 1966–2017

The demersal resources analyzed included bony fish, cephalopod, and crustaceans species. The fish group comprised of seven taxa: Hake (*Merluccius merluccius*), red mullets (*Mullus* spp.—i.e., *M*. *barbatus* and *M*. *surmuletus*), black seabream (*Spondyliosoma cantharus*), pandoras (*PagelIlus* spp.—mainly *P*. *erythrinus*), red porgy (*Pagrus pagrus*), rockfish (Scorpaenidae), and common sole (*Solea solea*); the cephalopods group of four taxa: European squid (*Loligo vulgaris*), shortfin squid (*Illex coindetii*), cuttlefish (*Sepia officinalis*), and octopus (*Octopus vulgaris*), and the crustacean group of one taxon of combined shrimp species (mostly deep water rose shrimp—*Parapenaeus longirostris* and caramote prawn—*Melicertus kerathurus*). The period of data used spans more than half a century (from 1966 to 2017; Figure 2).

### 2.3 Statistical analyses

The IRA framework was used to study the multivariate response of the demersal resources (“system”) to environmental change (“stressors”) and carry out a resilience assessment (Vasilakopoulos & Marshall, 2015; Vasilakopoulos et al., 2017). The IRA is an extension of the IEA framework (Levin & Möllmann, 2015; Möllmann & Diekmann, 2012; Möllmann et al., 2009). On top of the IEA routines used to study the multivariate development of complex natural systems, the IRA applies non‐additive modeling on the system‐stressor relationship and quantifies ecological resilience to construct stability landscapes.

As a first step, we reduced the complexity of the multivariate datasets by means of a multivariate analysis (principal component analyses, PCA) and implemented a suite of IEA methods to identify regime shifts (chronological clustering, breakpoint analysis, STARS). The analysis was applied to the biological system and environmental stressor datasets separately, to find holistic indicators of both the system's and stressors’ state and identify possible alternate regimes. Subsequently, to investigate the system‐stressor relationship, the system holistic indicator was regressed against the stressors’ indicator using additive (continuous) statistical models (GAMs). The fit of the GAM models was then compared to the respective fit of non‐additive (discontinuous) statistical models (TGAMs) to assess the type (continuous/discontinuous) of the system response and estimate the potential alternate attractors and threshold years of the system. Finally, a resilience assessment was carried out and a stability landscape was constructed. The analysis was carried out in R (R Core team, 2019) using packages *energy* (Rizzo & Szekely, 2018—correlation analyses), *vegan* (Oksanen et al., 2018—PCA), *rioja* (Juggins, 2017—Chronological Clustering), *mgcv* (Wood, 2017—GAMs/TGAMs), *strucchange* (Zeileis et al., 2002—breakpoint structural analysis), and *akima* (Akima, 1978—folded stability landscape); apart from STARS, which was applied using a stand‐alone Excel add‐in downloaded from https://www.beringclimate.noaa.gov/regimes/ (Rodionov, 2004).

#### 2.3.1 Principal component analyses

To investigate the multivariate temporal development of the “system” and the “stressors,” two separate PCAs were conducted based on the LPUC of the 12 demersal species/stocks (PCAsys) and the 12 environmental variables (PCAstr), respectively (e.g., Litzow et al., 2014; Möllmann & Diekmann, 2012; Möllmann et al., 2009). PCA converts correlated variables into linearly uncorrelated variables (principal components – PCs) through orthogonal transformation of data to a new coordinate system. PCA ranks PCs best explaining overall variation allowing comparison of trends in the population PC‐scores with trends in the stressor PC‐scores (Möllmann & Diekmann, 2012). To identify the key variables driving the system and stressor trends through time, the contributions of all original variables on PC‐scores (loadings) along the first and second principal component axes (PC1 and PC2) were calculated for PCAsys and PCAstr. Further details on PCA, the mathematical background and its interpretation can be found in Rao (1964) and Legendre and Legendre (1998).

The temporal development of each variable in the data set was visualized using the traffic light framework (Choi et al., 2005; Kenny et al., 2009; Möllmann et al., 2009). For this, variables were sorted according to their loadings on the PC1 axis, raw values of each variable were categorized into quintiles, and each quintile was given a specific color. For each variable, values of the lowest and highest quintile were indicated in green and red, respectively, with another three levels of intermediate colors.

#### 2.3.2 Chronological clustering

Chronological Clustering is a variation of cluster analysis, designed to run on ordered/successive samples like time‐series (Andersen et al., 2009; Legendre et al., 1985). The method calculates a similarity matrix combining samples into clusters, based on a permutation test and predefined significance and connectedness levels (Legendre et al., 1985). In our case, chronological clustering was applied using the "coniss" method (Constrained Incremental Sums of Squares—Grimm, 1987) on the 12 demersal species/stocks LPUC time‐series, as well as on the 12 environmental variables.

#### 2.3.3 Breakpoint structural analysis

Breakpoint analysis (Zeileis et al., 2002) allows identifying statistically significant changes in the mean level of subsets of a time series. A time series is randomly split in two or more subsets (‘‘data windows’’) and the mean levels are compared through a modified *F* test (‘‘structural change’’ or *sctest*—Zeileis et al., 2010). The procedure is repeated iteratively until all significant breakpoints (if any) are identified. The Bayesian Information Criterion is used as an objective criterion to determine the number of breakpoints and their associated dates with the corresponding 95% confidence intervals. In this study, breakpoint structural analysis was applied to the first two principal components of PCAsys and PCAstr to assess the year(s) of statistically significant change. All analyses were performed in R library *strucchange* (Zeileis et al., 2002).

#### 2.3.4 STARS

The Sequential t‐Test Analysis of Regime Shifts (STARS) is based on a sequential Student's *t*‐test that signals the possibility of a regime shift (Rodionov, 2004); the identified year of regime shift, is indicated by the calculated probability level. The method consists of calculating a *Regime Shift Index* (RSI), to accept or reject the hypothesis of a regime shift at each observation. RSI represents a cumulative sum of normalized anomalies relative to a critical value (Rodionov, 2006). The time scale of shifts to be considered is controlled primarily by the cut‐off length, which is similar to the cut‐off point in low‐pass filtering, and determines the minimum length of the regimes for which the magnitude of the shifts remains intact (Rodionov & Overland, 2005). A longer cut‐off length hence identifies the strongest signal (as opposed to many smaller events). Herein, STARS was applied to detect significant shifts in the time‐series of the first two principal components of PCAsys and PCAstr during 1966–2017 using a cut‐off length of 15 years. A tool for the detection of a regime shift based on STARS is available on the website http://www.beringclimate.noaa.gov/regimes.

#### 2.3.5 TGAMs

TGAMs are special cases of GAMs (Generalized Additive Models—Hastie, 2017). GAMs assume additive and stationary relationships between the response and explanatory variables, while TGAMs can represent an abrupt change in the relationships between the response and explanatory variables (i.e., a threshold in a specific year) signifying that a relationship is best described by two different functions, one before and one after the threshold (Casini et al., 2009; Ciannelli et al., 2004). These different functions are visualized as separate response curves (TGAM branches).

Threshold GAMs were used to regress environmental variables against biological variables (LPUC) and identify different response “regimes.“ To compare the fit of continuous (GAMs) and discontinuous models (TGAMs) on the relationships between the system indicator PC1sys and environmental stressors PC1str, at different time lags, the “genuine” cross‐validatory squared prediction error (gCV) was computed (Ciannelli et al., 2004). The gCV accounts for the estimation of the threshold line and the estimation of the degrees of freedom for the functions appearing in all additive and non‐additive formulations. In TGAMs, the threshold years were selected by minimizing the generalized cross validation (GCV) of the whole model through the implementation of a searching algorithm which, in our case, runs the model for 50 possible threshold years between the 0.2 lower and the 0.8 upper quantiles of the time series.

It should be noted that while chronological clustering, breakpoint analysis, and STARS detect shifts in the relationships between each of the holistic system and/or stressor variables and time, TGAMs detect shifts in the relationship between the holistic system and a stressor variable. Hence, it is possible to have shift(s) in the system and/or stressors identified by chronological clustering/breakpoint analysis/STARS, but no shift in the system‐stressor relationship (in which case a GAM would provide a better fit than a TGAM), and vice versa. For example, if the system shifts at the same time when the stressors shift, then the system‐stressor relationship could be linear. However, a shift in the system that coincides with a shift in the system‐stressor relationship, suggests a system shift, that is, a discontinuous response to changing stressors.

#### 2.3.6 Resilience assessment

Following the IRA framework (Vasilakopoulos et al., 2017), the branches of the optimal TGAM model(s) were considered to represent the alternate attractors of the system, forming fold‐bifurcations. The plausibility of the transition trajectories (i.e., shifts happening at local extrema of stressors followed by hysteresis) was then confirmed (Tsimara et al., 2021). Resilience is expected to increase with increasing distance of a system state to the tipping point (“horizontal component” of resilience—*hComp*; Figure S0). In contrast, resilience is expected to decrease as the distance of the system state to its attractor increases (“vertical component” of resilience—*vComp*; Figure S0). The annual resilience estimate (*Res*_y_) was thus the sum of the horizontal distance of each system state (represented by “year”), from the tipping point of its regime (*hComp*_y_) minus the vertical distance of each year from its fitted attractor (*vComp*_y_), facilitated by the fact that both the x‐ (PC1str) and y‐axis (PC1sys) were on the same scale (i.e., no standardization was needed). To calculate the position of the tipping point(s) of each regime along the trajectory of their respective attractor, the x‐coordinates of the tipping points were set to allow all *Res*_y_ estimates within each regime to be non‐negative. *Res*_y_ was scaled by dividing with the maximum value observed to calculate relative resilience (*rRes*_y_). The folded stability landscape emerged through linear interpolation of all *rRes*_y_ values onto a 100 × 100 grid, using the “akima” package in R. More details on the IRA methodology are provided in Vasilakopoulos et al. (2017).

Due to each of the methods employing a different terminology to identify the years when a shift occurred (e.g., marginal year, change‐point, shift year), hereafter when we refer to the timing of shifts we use the term “threshold year,” referring to the last year of the old regime, for all methods.

## 3 RESULTS

### 3.1 Environmental “stressors”

**FIGURE 3:**
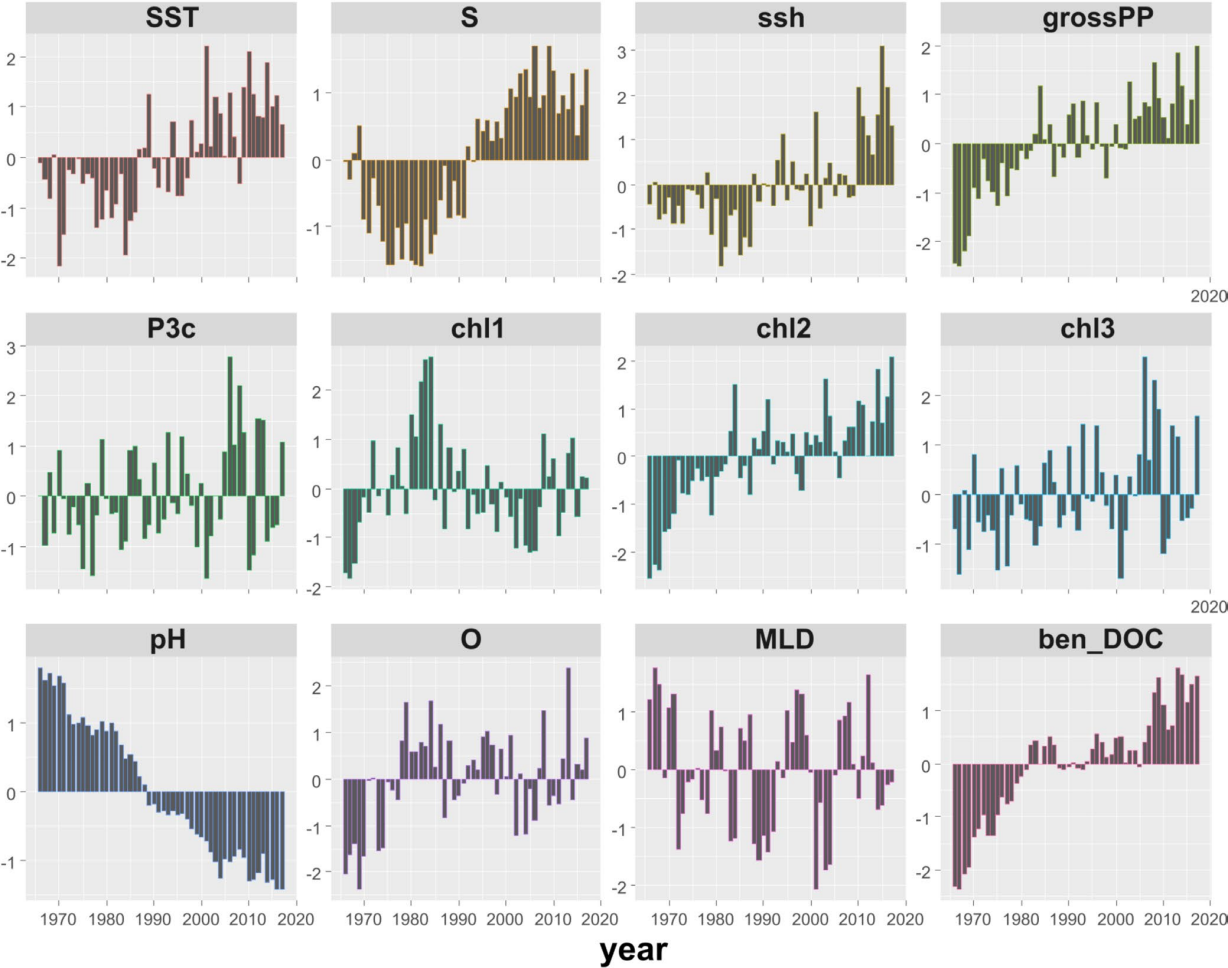
Anomaly of environmental variables expressed as difference from the overall mean divided by the standard deviation in the Aegean Sea during 1966–2017

Visual inspection of the anomalies (Figure 3) and traffic light plots (Figure S4 top), illustrating the temporal changes of all stressor variables, suggested that most variables exhibited coherent trends in the period 1966–2017. Most prominently, pH exhibited a continuous decrease, while productivity‐related variables (ben_DOC, grossPP, chl2) exhibited a continuous increase.

**FIGURE 4:**
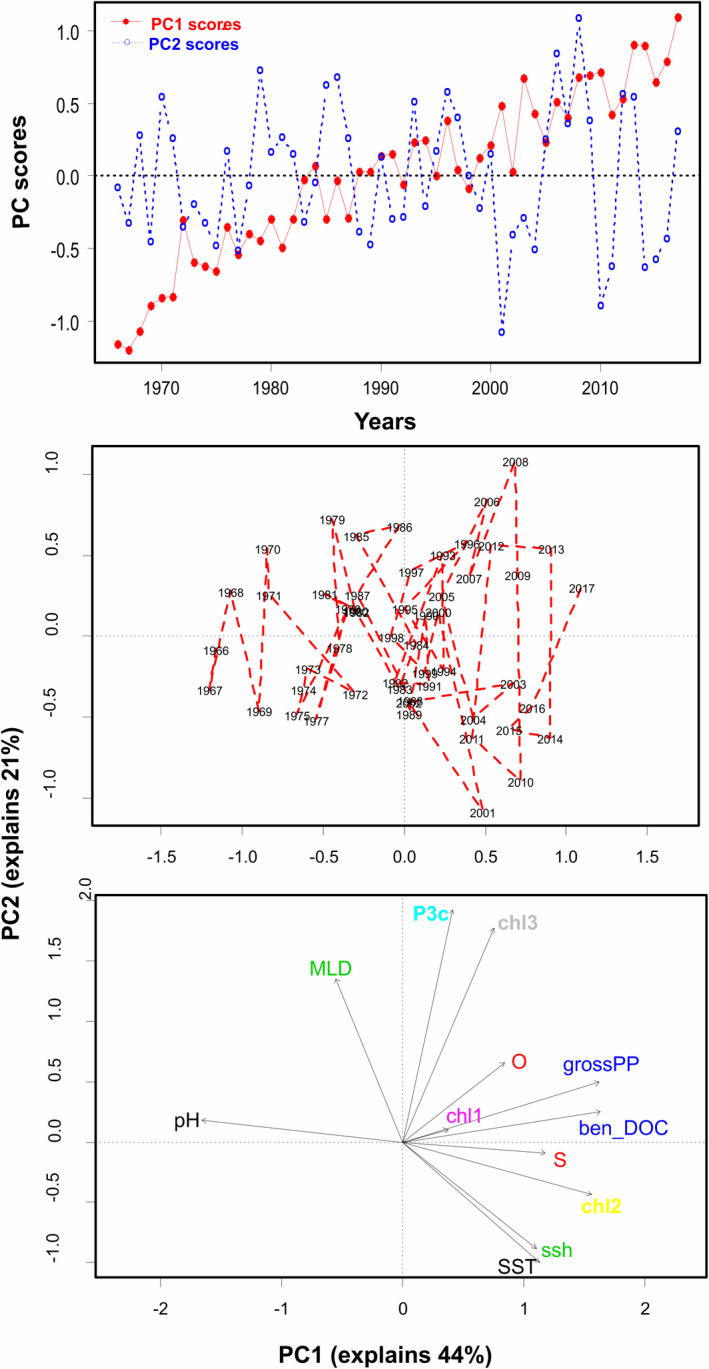
PCA of the stressors (environmental system). Annual PC‐scores for the first two principal components of PCAstr versus time (top), time trajectory of PC1 versus PC2 of PCAstr (mid), and loading plot of environmental factors in PCAstr (bottom). PCAstr conducted for the Aegean Sea environmental indices covering the period 1966–2017

Based on the PCAstr outputs, the first two PCs (PC1str and PC2str) explained a relatively high proportion of the total variability: 44% and 21%, respectively. Based on the PC loadings, grossPP with pH, and P3c with chl3, were the most important variables across the PC1str and PC2str axis, respectively (Figure 4 bottom). The multivariate temporal development of the stressors during this period was visualized by plotting the annual trends of both PC1str and PC2str (PC‐scores vs. time—Figure 4 top) and PC scores of each year along the PC1str and PC2str axes on a two‐dimensional system (Figure 4 mid). A gradual transition along the x‐axis during 1988–1992 was apparent; this transition was due to the contrasting loadings of different stressors on PC1str (Figure 4 bottom).

**FIGURE 5:**
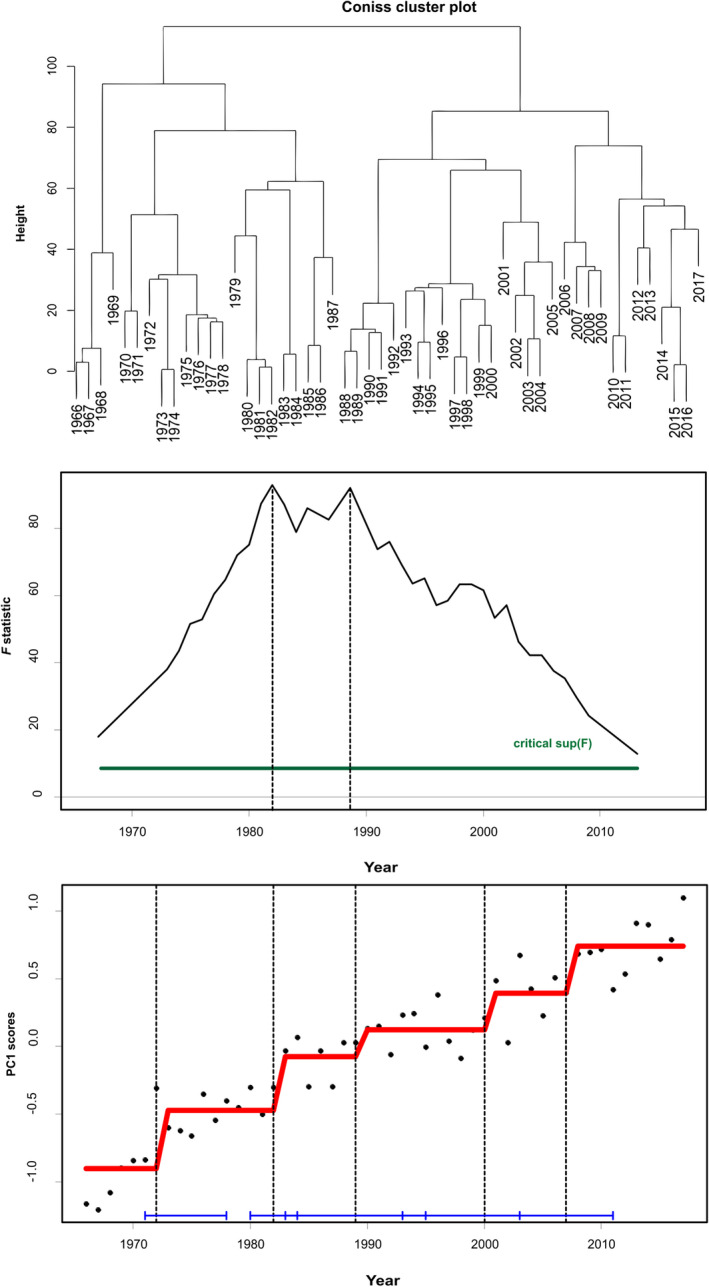
Environmental time series from the Aegean Sea during 1966–2017 analyzed by chronological clustering (top) and breakpoint analysis by sequential *F* tests (mid) on the first principal component PC1str (bottom). Critical *F* levels (green lines) and fitted mean values before and after the identified change‐point (red lines)

Chronological clustering and the corresponding dendrogram (Figure 5 top) suggested two main regimes (before and after 1987). Breakpoint structural analysis and sup(*F*) statistic confirmed that the presence of multiple change‐points in PC1str is statistically well supported (Figure 5 mid and bottom), apparently capturing the existence of alternate stepwise changes. In terms of statistical significance, 1982 and 1988 threshold years were stronger than the 1972, 1995 and 2007 ones.

**FIGURE 6:**
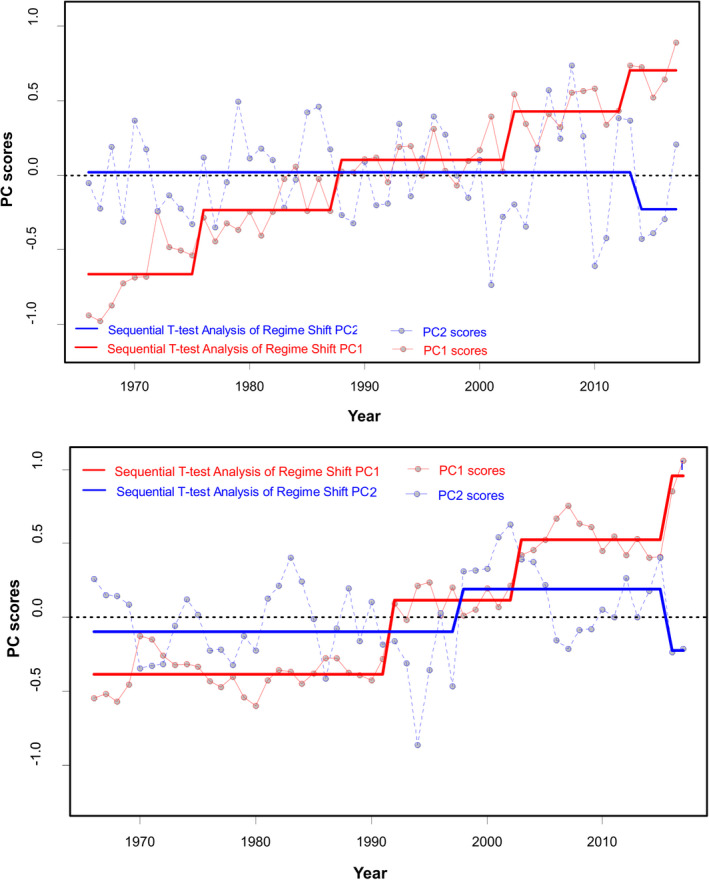
Results of the regime shift analysis (STARS) on the principal component analysis: (top) PCAstr output: regime shift years identified in time series of PC1str and PC2str scores of the environmental time series from the Aegean Sea during 1966–2017 (bottom) PCAsys output: regime shift years identified in time series of PC1sys and PC2sys scores of the LPUC time series of demersal 12 species/taxa from the Aegean Sea during 1966–2017. Dots represent the scores of the two principal components; solid lines represent the stepwise trend showing the regime shift in the mean detected by STARS method using significance level *p* = .05 and a cut‐off length of 15 years

STARS identified four step‐changes (Figure 6 top), with the dominant being in the late 1980s, as reported by the respective Regime Shift Indices‐RSI (0.96, 1.29, 0.76, and 0.33 for 1976, 1988, 2003, and 2012, respectively). However, successive regimes were not very distinct, as there was a high degree of overlap amongst them (Figure 6 top).

### 3.2 Biological “system”

In terms of historical biomass trends (expressed as LPUC), three types of patterns were observed (Figure 3): (a) an increase up to mid‐1990s and a subsequent decrease afterwards for black seabream, common soles, and *Mullus* spp.; (b) an increasing trend since the 1960s for shrimps, squids, octopus, cuttlefish, red porgies, and hake; and (c) fluctuating trends for *Pagellus* spp. and rockfish. Traffic light plots (Figure S4 bottom), suggested that most demersal species followed conspicuous trends, with the late 1980s–early 1990s being the point in time where the first major changes in the configuration of the system occurred, with a period in the early 2000s exhibiting additional changes.

**FIGURE 7:**
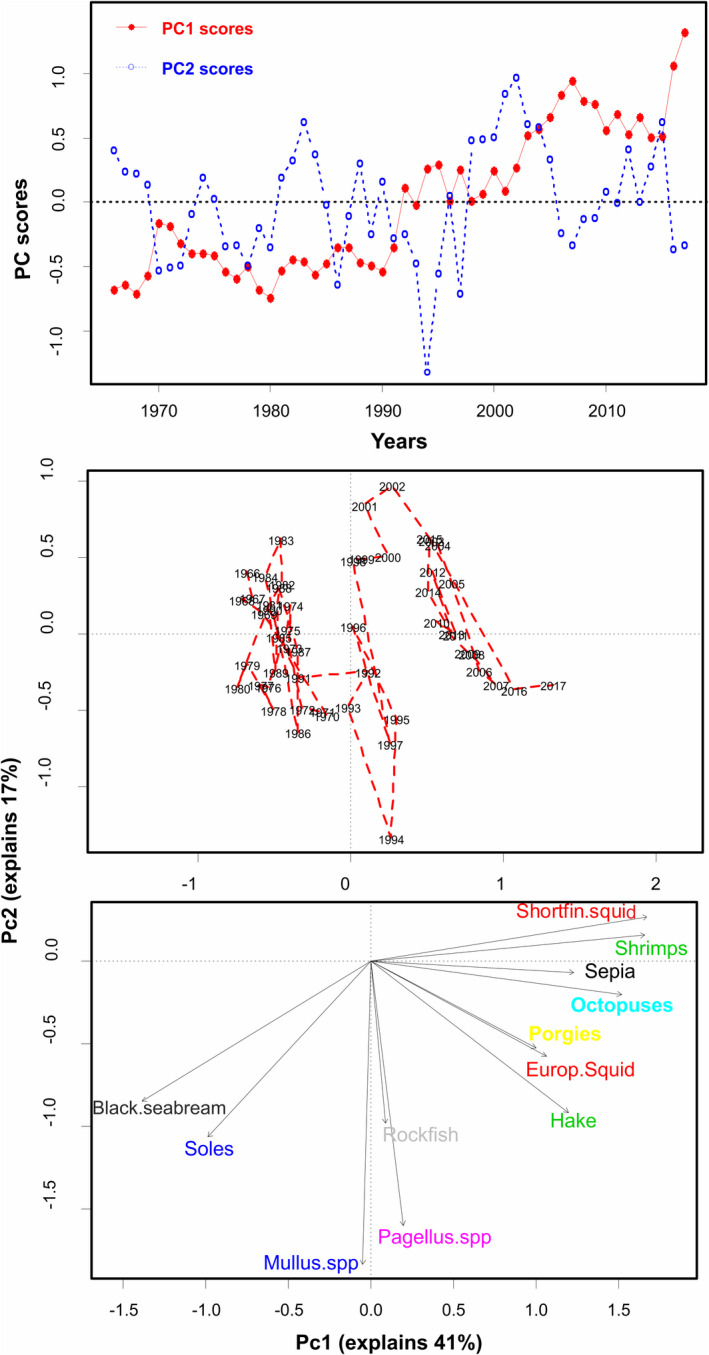
PCA of the biological system. Annual PC‐scores for the first two principal components of PCAsys versus time (top), time trajectory of PC1 versus PC2 of PCAsys (mid), and loading plot of demersal taxa LPUC in PCAsys (bottom). PCAsys conducted for the Aegean Sea demersal species covering the period 1966–2017

PC1sys and PC2sys explained 41% and 17% of the variation, respectively, with cephalopods and shrimps having the highest loadings across the primary axis (Figure 7). The progressive development of the demersal biological “system” is depicted through the annual trend of PCsys scores versus time (Figure 7 top) and the projection of the “trajectory” of the system along PC1sys (mostly) and PC2sys axes (Figure 7 mid and bottom). Three periods are visually apparent with two shifts along the x‐axis in 1992 and 2003. The shifts were mainly driven by a decrease in black sea bream and soles (high negative loadings) and an increase in cephalopods and shrimps (high positive loadings). The magnitude of change was considerable, being more than 50% for all the aforementioned species/taxa during the studied period.

**FIGURE 8:**
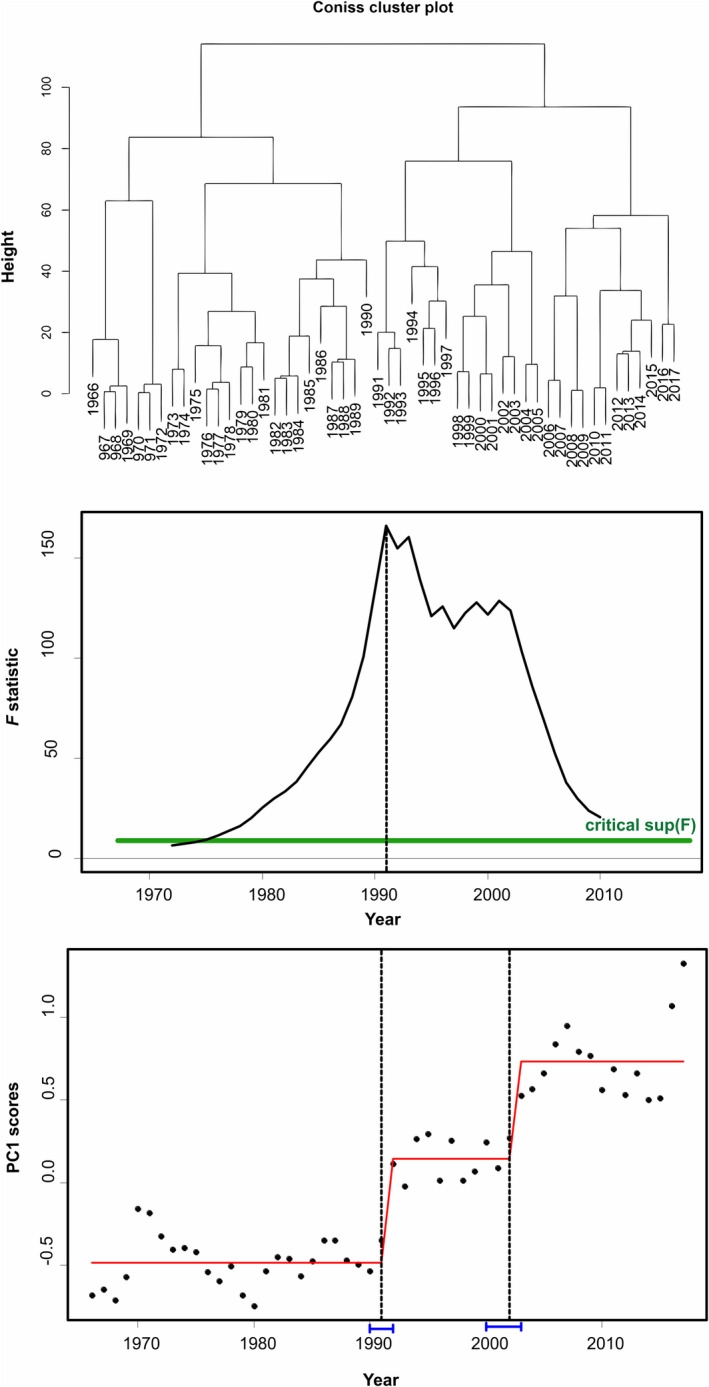
LPUC time series from the Aegean Sea during 1966–2017 analyzed by chronological clustering (top) and breakpoint analysis by sequential *F* tests (mid) on the first principal component PC1sys (bottom). Critical *F* levels (green lines) and fitted mean values before and after the identified change‐point (red lines)

Two main clusters were suggested by the dendrogram of the Chronological clustering analysis (Figure 8 top), with 1990 being the marginal/threshold year. Breakpoint structural analysis and sup(*F*) statistic confirmed 1991 as the change‐point in PC1sys (Figure 8 mid and bottom). A second change‐point in 2002 was also evident.

STARS detected significant shifts in the mean values of PC1sys, identifying three step‐changes (Figure 6 bottom), with 1992 supported mostly by the respective RSI (2.11, 1.05, and 1.22 for shift years 1992, 2003, and 2016 respectively). The three states in PC1sys (1966–1991, 1992–2002, 2003–2015) were very distinct and did not overlap, contrary to the states detected in PC1str (Figure 6). We cannot be conclusive regarding the last shift (2016), because STARS tends to detect false regime shifts in the last cut‐off window (Stirnimann et al., 2019).

### 3.3 System‐stressors relationship

**FIGURE 9:**
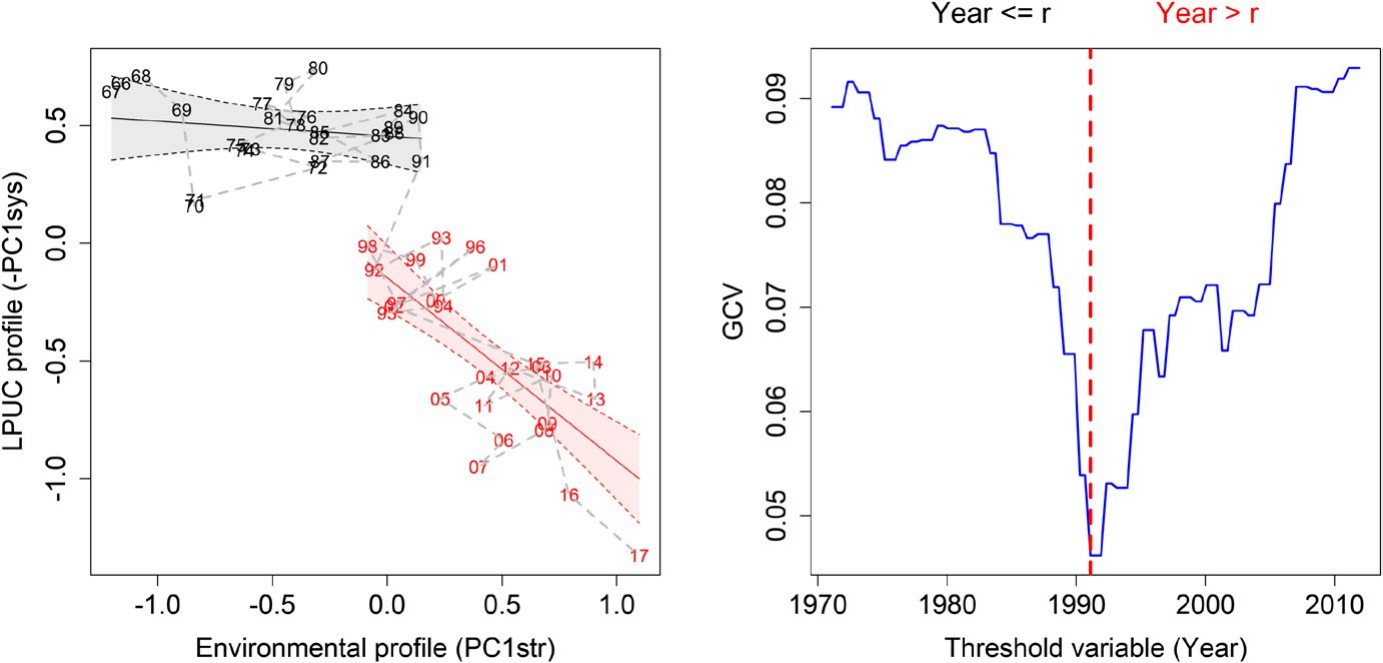
Threshold GAM of PC1sys (system) against PC1str (stressor) at lag 0 with the two identified regimes shown in gray (before 1991) and red (after 1991) (left) and profile of the GCV score as a function of the threshold variable (Year) with the dotted vertical red line representing GCV estimated at the threshold year (1991)

TGAMs offered superior fits to GAMs when modeling the regression of the biological “system” indicator (PC1sys) against the environmental “stressors” (PC1str) (Table 2). In all different time lags investigated, 1991 was found to be the threshold year. The 0‐lag model was found to provide the best fit amongst the tested TGAMs, but the difference in gCV between models with different lags was quite small (Figure 9; Table 2), especially those with a 0‐ and 1‐year lag. TGAMs can only detect a single threshold in the relationships between the response and explanatory variables. However, after the 1991 shift, years 1992–2002 and 2003–2016 formed two distinct clusters, with year 2017 being far from both (Figure 9). This hinted at the existence of two regimes in 1992–2016; hence, the fits of a GAM and a TGAM on the relationship between PC1sys and PC1str in 1992–2016 were also compared (Vasilakopoulos et al., 2017). The fitted TGAM (gCV = 0.0369) was found to provide a better fit than its respective GAM (gCV = 0.0628), indicating a second discontinuous response in the Aegean system with 2002 as a threshold year (Figure 9). Overall, the TGAM fits revealed twofold‐bifurcations with three alternate attractors in the biological system.

**TABLE 2:** The gCV and percentage of deviance explained (in brackets) of GAMs and TGAMs fitted on the relationships between PC1sys and PC1str, at 0‐ to 2‐year lags

PCAstr lag	GAM gCV	TGAM gCV	Threshold year
0‐lag	**0.0923 (74%)**	**0.0544 (87%)**	1991
1‐lag	0.0976 (72%)	0.0632 (86%)	1991
2‐lag	0.1002 (70%)	0.0745 (83%)	1991

Note

Bold font indicates the models with the lowest gCV values (optimal models). GAMs and TGAMs were formed by regressing the multivariate population index (LPUC) against the multivariate stressor index (physicochemical variables) in the Aegean Sea during 1966–2017.

The outcomes of all the methods employed herein are summarized in Table 3. There is a striking convergence of all approaches to almost the same years suggested as threshold years; an indication of a clear signal in the data that two regime shifts occurred in the system as a discontinuous response to changing environmental stressors.

**TABLE 3:** Estimated threshold years of regime shifts (i.e., last year of the older regime) identified by the methods employed on the Aegean Sea environmental (stressors) and demersal taxa (system) series during 1966–2017

Method	Estimated threshold year(s)	
	Environmental “stressors”	Biological “system”
Chronological clustering	1987 (secondary cluster split at 1969)	1990 (secondary cluster split at 2005)
Breakpoint analysis	1982, 1988	1990
STARS	1975, 1987, 2002, 2012 (high overlap)	1991, 2002 (no overlap)
TGAMs (PCA1sys ~ PCA1str)	1991, 2002	

Note

First three methods identify shifts in the relationship of the composite environmental “stressors” and biological “system” and against time, while TGAM identifies shifts in the relationship between the biological “system” and environmental “stressors.”

### 3.4 Folded stability landscape

**FIGURE 10:**
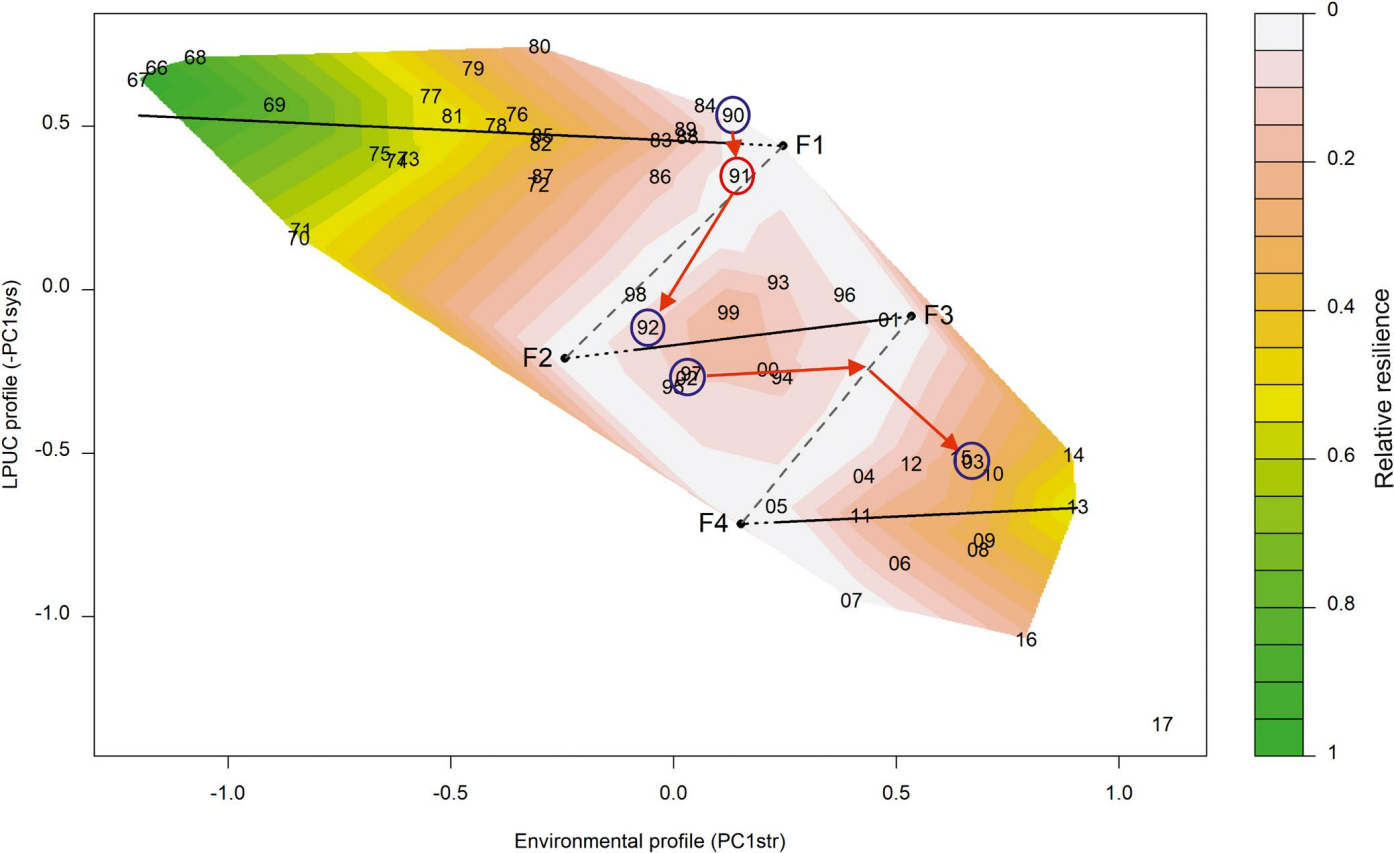
Empirical folded stability landscape with transition pathways of the Aegean demersal landings assemblage (“LPUC profile”) in response to environmental change during the period 1966–2017. The folded stability landscape exhibits two basins of attraction and four tipping points (F1, F2, F3, F4). Color scale indicates the relative resilience (“altitude”), continuous lines indicate the attractors, dotted black lines indicate the possible extensions of the attractors, and gray dashed lines indicate the borders of the basins of attraction. The prefix 19‐ and 20‐ of the years is omitted. Year 2017 has not been assigned to any basin of attraction as it differed substantially from its preceding years

The resilience assessment allowed the construction of a folded stability landscape of the biological system that exhibited three basins of attraction (regimes) (Figure 10). It should be noted that the area between tipping points (F1 and F2; F3 and F4) hosts barely any observations, as it is an area of repulsion; this is a common feature of stability landscapes. Hence, the linear interpolation of resilience between the years of the filled contour often creates “corridors” of seemingly higher resilience between years with high resilience belonging to different states (Vasilakopoulos et al., 2017). To correct for this, we added two theoretical “years” (not plotted) between F1 and F2, and between F3 and F4, whose resilience was calculated based on their distance from the attractors and tipping points, exactly like for all other years.

The folded stability landscape suggested that the system retained its original state for 25 years despite concurrent large changes in the environment. The system even absorbed two environmental step‐changes without changing its state, owing to its high resilience. However, the system's resilience was gradually eroding in response to environmental change, and in 1990–1992, a small perturbation, while it was lying in an area of low resilience, led it abruptly to a new state through “vertical movement,” in line with the theory of critical transitions (Scheffer, 2009) (Figure 10). This new state was retained for 10 years (1992–2002) in light of medium environmental change, even exhibiting some hysteresis. This hysteresis was exhibited as a retention of the system at the new PC1sys levels, despite a reversal of PC1str to pre‐shift levels during the second half of the 1990s. However, another large step‐change in the environmental stressors during 2002–2003 pushed the system into a new state through “horizontal movement” (Scheffer, 2009). The system exhibited some hysteresis in that third state too, but toward the end of the time‐series (in 2016/17) exhibited another change. Year 2017 was particular, because both its system and stressor levels were outside the ranges observed in 1966–2016 (Figure 10). Therefore, we decided to plot it on the stability landscape but not to include it in the calculation of the basins of attraction, as it remains uncertain whether it is part of the last state or part of an entirely new state. Future data from subsequent years may clarify this.

## 4 DISCUSSION

A combination of multivariate analyses, non‐additive modeling, and a resilience assessment, that is, an IRA, was employed to investigate a plausible effect of CC on the demersal resources of the Aegean Sea (east Mediterranean Sea) over the past six decades. This analysis revealed that pronounced changes have occurred in both the environment and the biological system over the studied time period. It is suggested that the biological system has exhibited a discontinuous response to CC, with two climate‐induced regime shifts occurring in the past 25 years. There is evidence for twofold‐bifurcations and four tipping points in the system, forming a folded stability landscape with three basins of attraction (Scheffer, 2009).

The majority of the environmental stressors investigated exhibited strikingly increasing trends (temperature, salinity, primary production indices) with the late 80s–early 90s being the point where values started exceeding the global historical mean. Zervakis et al. (2000) suggested that the drastic changes in eastern Mediterranean climatic regime in the early 1990s΄ is attributed to a process initiated in the North Aegean in the winter of 1986/1987 and was intensified by another formation event in 1992/1993. Conversi et al. (2010), provide evidence that the north/northwestern Mediterranean Sea underwent a major change at the end of the 1980s that can be considered a regime shift; the phenomenon is more likely linked to a wider northern hemisphere change. Two major climatic shifts have been documented in the northern hemisphere during the 20th century, in the mid‐1970s and the late 1980s. Both were related to a change of the NAO Index and associated with an increase of temperature. Concurrent with these climatic shifts, several marine ecosystems of North Europe (North Atlantic, North and Baltic Seas, Scotian shelf and Black Sea) and the Mediterranean Sea were reported to have undergone ecosystem regime shifts (Fortibuoni et al., 2017; Möllmann & Diekmann, 2012). We also detected environmental regime shifts in the Aegean Sea both in the mid‐1970s and in the late 1980s, followed by additional shifts in the early 2000s and early 2010s. However, all these shifts were not associated with fully separated states, as there was always some degree of overlap between successive states.

The biological system in the Aegean Sea, as inferred through historical fishery dependent relative biomass trends of demersal taxa, also exhibited marked changes. Two regime shifts were observed in the early 1990s and early 2000s, which resulted in three fully separated states. Cephalopods and crustaceans increased throughout the study period, while fish showed diverse trends (increasing: red porgy and hake; decreasing: sole and black seabream; fluctuating: red mullets, pandoras and rockfish). Cephalopod expansion has already been identified and linked to CC. Pinnegar et al. (2017), reporting on the increasing cephalopod catches in the UK, point out that squid has replaced the region's traditional target species, such as haddock and cod. Doubleday et al. (2016) demonstrated that cephalopod populations have increased globally over the last six decades; a result, that is, remarkably consistent across a highly diverse set of cephalopod taxa. van der Kooij et al. (2016) identified significant positive relationships when comparing squid distribution and abundance with key climatic variables. Squid (*Loligo vulgaris*) is projected to expand northwards over the next 40 years with habitat suitability in UK waters increasing by around 31% (Defra, 2013). Tzanatos et al. (2014), examining fisheries landings fluctuations for 59 commercial species in the Mediterranean Sea, detected significant year‐to‐year correlations with temperature for nearly 60% of the cases; the majority being negatively related and showing declining catches. Increasing trends were found, mainly for species with short life spans, apparently benefitting from the increase in water temperature (Tzanatos et al., 2014). Vasilakopoulos et al. (2017) examined the temporal development of the Mediterranean fisheries landings in response to sea warming during 1985–2013, and concluded that a nonlinear tropicalization of the Mediterranean Sea has occurred, with the Mediterranean biota being increasingly dominated by thermophilic species. This tropicalization had also functional consequences for the Mediterranean ecosystems, as indicated by the establishment of new biological traits (Tsimara et al., 2021). Our findings for the Aegean Sea are also in line with other previously identified effects of warming in Mediterranean ecosystems, such as: elevated MTC (Cheung et al., 2013; Tsikliras & Stergiou, 2014), changes in the biology, abundance and range of different species (Marbà et al., 2014) and ecosystem regime shifts in the marine communities of the eastern and western Mediterranean Sea and the Northern Adriatic Sea (Fortibuoni et al., 2017).

Intriguingly, the shape of the stability landscape for the Aegean Sea's biological system suggests that while the initial state (before 1991) was rather resilient to CC this was not the case with the two subsequent ones (intermediate state: 1992–2002; recent state: 2003–2016). The initial state lasted for 25 years and absorbed two environmental step‐changes retaining its original configuration, albeit paying the price in terms of resilience erosion. While the early 1990s regime shift was associated with a small perturbation at a time when the system had low resilience, the next environmental step‐change in the early 2000s pushed the system immediately into a new state. In other words, the intermediate and recent states were more “narrow” in terms of accepted environmental conditions (x‐axis value range in Figure 10) compared to the initial state. It could be that environmental change, up to 1991, represented “familiar” conditions that had occurred during the evolutionary history of the biological system; hence, the system had built some resilience to them. However, after 1991, the environment might have moved to areas never experienced before; hence, the system struggled to stabilize within its new states. The likely onset of a new state in 2016/17 corroborates this hypothesis. The biological system seems to be increasingly prone to discontinuous regime shifts in response to CC over the past 25 years as it is entering areas of unprecedented climatic conditions. This is an important finding that might be also relevant to other marine areas moving into novel climatic regimes as a result of the global CC.

At a global scale, changes in marine fisheries catch have been documented to be related to changes in ocean features (e.g., temperature), with an increasing dominance of catches of warmer waters species at higher latitudes and a corresponding decrease in the proportion of catches of subtropical species in the tropics (Cheung et al., 2013). Marine biota responds to ocean warming through changes in distribution, abundance, phenology, and body size, leading to alteration of community structure and trophic interactions ultimately affecting fisheries catches (Cheung et al., 2010). In general, species and marine communities respond differently to change, depending on the organisms’ capacity to adapt to novel regimes. Lower trophic level organisms are more able to integrate seasonal thermal changes, whereas, higher trophic level species depend on both the availability of lower trophic level organisms and environmental conditions (Friedland et al., 2019).

The Mediterranean Sea is a semienclosed basin, sensitive to bottom‐up processes (Piroddi et al., 2017), where the effects of CC are likely to be more pronounced and become apparent sooner than in other, more open ocean regions (Giorgi, 2006). The Mediterranean basin is projected to be a hotspot of CC in terms of both warming and an increase in extreme events (Calvo et al., 2011; de Madron et al., 2011; MEDCLIVAR, 2004). Since the late 1990’s, the abrupt increase in temperature has facilitated the establishment and northward migration of warm‐water species from adjacent seas at an unexpectedly rapid rate (Raitsos et al., 2010).

Within the Mediterranean, the Aegean Sea in particular, encompasses certain peculiarities that may categorize it among the most vulnerable regions: an extended length of coastline (12,000 km—almost 1/4 of the whole Mediterranean coastline), complex bathymetry, and numerous islands (more than 3000 islands or islets), hosting various intensive anthropogenic activities such as industry, aquaculture, maritime shipping (over 35,000 vessels per year traversing through its waters), and tourism (e.g., 800,000 yachts sailing in the Mediterranean, Aegean Sea being one of the most popular sailing destinations), (Sakellariou et al., 2005; Sofianos et al., 2002). However, its most striking characteristic is the largest fishing fleet in Europe (~12,000 vessels, out of a total of 15,000 in Greece and 80,000 in the EU)^1^, mostly comprising of small‐scale fishery vessels, operating in a traditional manner and being multi‐specific in nature (HELSTAT, 1964–2017). Adding to this vulnerability, the region has been probably extensively exploited by humans earlier than any other marine region of the world (NHRF, 2010). The most recent warning sign comes from the acceleration in the annual mean rate of warm and tropical alien species introduced (a 150% increase of species entry in the last two decades), with the speed of alien species spreading being faster than environmental change (EASIN, 2020; Raitsos et al., 2010). In the Mediterranean, Aegean Sea is now considered among the regions being hot‐spots for alien species (EASIN, 2020; Zenetos et al., 2011).

The potential vulnerability of the fishing sector in the Aegean Sea to the effects of CC needs to be better understood and considered to inform the development of effective management and adaptation policies. Free et al. (2019) highlight the importance of accounting for CC in fisheries management: out of 235 populations investigated from 38 ecoregions worldwide it was deduced that, besides fishing, an additional factor that led to a decrease in maximum sustainable yield (MSY) was CC. MSY decreased, as a result of CC, by 4.1% from 1930 to 2010, with five ecoregions experiencing losses of 15%–35%. On the other hand, the progressive establishment and colonization of thermophilic species in new areas and the expected changes in migratory behaviors could create new fishing opportunities in the whole basin (Lloret et al., 2015). Future projections for the Mediterranean predict that the western basin is expected to become more oligotrophic with a surface density decrease, while surface production is expected to increase in the eastern basin linked to a density increase (Macias et al., 2015). Regional changes in fish abundance and their distribution will alter species richness, with an expected increase in overall richness by the mid‐21st century in the Eastern Mediterranean, and a decrease in the western region (Albouy et al., 2013). This means that, while the biological transitions in the Aegean Sea detected here are of profound importance for local fisheries management, which should be tailored to the most recent ecosystem state, we should also prepare for a future where abrupt ecosystem shifts to novel states will become the norm. Consequently, managers and fishers need to focus on increasing the adaptive capacity of the fishing sector to protect its socio‐economic resilience in the context a decreasing ecological resilience. This task could encompass diversification of the fishing activities, adjustment of the timing and location of spatio‐temporal measures, as well as new market regulations.

While our study has focused on the effect of CC, other potential stressors of the biological system may be at play, with the most important one being fishing. The absence of key fishing data for the most part of the time series (i.e., before 2000; e.g., actual fishing effort, stock status) hindered a deeper understanding of potential fishing effects. Nevertheless, a LPUC index was used as a proxy for biomass with vessel capacity (in GT) as a surrogate of fishing effort. Based on these limitations, certain assumptions had to be made: (i) fishery performance can approximate, reasonably, the relative biomass at sea (Marr, 1951), and (ii) commercial fishery statistics is a fishery performance index (Ricker, 1975). Obviously, other fishery effects (e.g., technological creep) and other external drivers may exist interacting in ways not fully identified herein.

The Greek fleet has undergone some dramatic changes during the study period; Greece joining the EU in the early 1980s, gave access to funds allowing for investing in and expanding the fleet. However, the EU Common Fisheries Policy reforms in 1992 and 2002 (EC, 2002a, 2002b; EEC, 1992) requesting adjustment of fishing capacity of the fleets, led to subsidies for mass decommissioning and scrapping of vessels. As a result, the Greek fleet contracted by more than 30% in just 20 years, from more than 22,000 vessels in 1995 down to almost 15,000 vessels in 2016 (Figure S5). Yet, the effect of fishing has been suggested as a driver of stock status only during the past two decades (e.g., Vasilakopoulos et al., 2014). Official evaluations conducted by the scientific groups responsible for assessing stocks in the Aegean Sea (EU STECF, GFCM) conclude that currently only hake can be considered overfished (GFCM, 2017, 2019). Red mullets and striped red mullets (*Mullus* spp.) may be within safe biological limits (GFCM, 2019; STECF, 2020), while all other demersal species considered herein remain with no advice or unassessed. As a result, historical overfishing of demersal resources in the Aegean Sea, prior to 2000, has not been satisfactorily documented; some efforts based only on landings data (e.g., Tsikliras et al., 2015) ignored fleet/effort evolution (taken into account in our study), and cannot elucidate the fishing effect on stocks in the past (1960s–1990s).

Ultimately, although fishing may have contributed to the observed shifts, the coupling observed in the dynamics of the biological and stressor systems provides strong evidence that CC has been a major driver and that the human factor alone could not lead to such a conspicuous event. The main shift identified herein (1992) occurred during a period that fishing capacity (and presumably fishing intensity) remained stable (1988–1996; Figure S5). This strengthens the argument that the key driver behind the observed system dynamics is CC rather than fishing. Nevertheless, it could be argued that this sensitivity of the system to CC was related to some extent to the overfished status of the fisheries resources (Hsieh et al., 2006). Further understanding of the interactions and synergies between CC and fishing, and the ways they affect different trophic levels and the ecosystem as a whole is needed.

In conclusion, there is strong evidence that the Aegean Sea demersal community has undergone a biological transition from one regime (pre‐1991) to another (2003–2016), after a prolonged intermediate state (1992–2002). It is suggested that this transition was a discontinuous response to CC. The demersal biological system shifted from a state where fish species dominated to a state dominated by cephalopods and crustaceans, in an environment characterized by elevated water temperatures, higher salinities and more productive and acidic waters. Furthermore, the system needs to be closely monitored as it moves toward an area of unprecedented environmental conditions; a *terra incognita*, which could entail further discontinuous responses to CC.

## CONFLICT OF INTEREST

None declared.

## AUTHOR CONTRIBUTIONS

**Dimitrios Damalas:** Conceptualization (lead); Data curation (lead); Formal analysis (lead); Investigation (lead); Methodology (lead); Software (lead); Writing‐original draft (lead); Writing‐review & editing (lead). **Vasiliki Sgardeli:** Conceptualization (lead); Data curation (lead); Formal analysis (lead); Investigation (lead); Methodology (lead); Software (lead); Writing‐original draft (lead); Writing‐review & editing (lead). **Paraskevas Vasilakopoulos:** Conceptualization (lead); Formal analysis (lead); Investigation (lead); Methodology (lead); Software (lead); Writing‐original draft (lead); Writing‐review & editing (lead). **Georgios Tserpes:** Writing‐original draft (supporting); Writing‐review & editing (supporting). **Christos Maravelias:** Funding acquisition (equal); Writing‐original draft (supporting); Writing‐review & editing (supporting).

## Supporting information

Supplementary Material

## Data Availability

Data on historical physicochemical variables for the Aegean Sea during the period 1966–2017 were made available through the Horizon2020 CERES project (http://ceresproject.eu/). Marine fisheries’ landings and fleet capacity data for the Greek fleet have been extracted from the yearly bulletins of the Hellenic Statistical Authority (HELSTAT, 1964–2017). All aforementioned data are uploaded on FigShare: https://doi.org/10.6084/m9.figshare.16860457.v1

## ENDNOTE

^1^
EU fleet register: https://webgate.ec.europa.eu/fleet‐europa/index_en
