# Supplementary material for: Evidence of climate‐driven regime shifts in the Aegean Sea’s demersal resources: A study spanning six decades
Source: Ecol Evol. 2021 Nov 13;11(23):16951–71. doi: 10.1002/ece3.8330 (PMC8668738; doi:10.1002/ece3.8330)
Supplement: Supplementary file 1 — Supplementary Material [file ECE3-11-16951-s001.docx]

**Annex – Supplementary material**

**Tables**

**Table S1**. Full list of environmental variables investigated

| **Variable name** | **Units** | **Description** |
| --- | --- | --- |
| T | degC | Temperature |
| S | psu | Salinity |
| U | m/s | Eastward velocity |
| V | m/s | Northward velocity |
| W | m/s | downward component of current velocity |
| ssh | m | Sea surface elevation |
| grossPP | tonsC/d | Gross Primary Production |
| netPP1 | mgC/m^3/d | Net Primary Production by diatoms |
| netPP2 | mgC/m^3/d | Net Primary Production by nanophytoplankton |
| netPP3 | mgC/m^3/d | Net Primary Production by picophytoplankton |
| netPP4 | mgC/m^3/d | Net Primary Production by microphytoplankton |
| P1c | mgC/m^3 | Diatoms C |
| P2c | mgC/m^3 | Nanophytoplankton C |
| P3c | mgC/m^3 | Picophytoplankton C |
| P4c | mgC/m^3 | Microphytoplankton C |
| Z4c | mgC/m^3 | Mesozooplankton C |
| Z5c | mgC/m^3 | Microzooplankton C |
| Z6c | mgC/m^3 | Heterotrophic Nanoflagellates C |
| B1c | mgC/m^3 | Bacteria C |
| chl1 | mg/m^3 | Diatoms Chl |
| chl2 | mg/m^3 | Nanophytoplankton Chl |
| chl3 | mg/m^3 | Picophytoplankton Chl |
| chl4 | mg/m^3 | Microphytoplankton Chl |
| pH | -log[H+] | pH |
| O | mmol/m^3 | Oxygen |
| ZPredPhyt | tonsC/d | Zooplankton Predation on Phytoplankton |
| ZPredBac | tonsC/d | Zooplankton Predation on Bacteria |
| ZPredPart | tonsC/d | Zooplankton Predation on Particulate Matter |
| ComProd | tonsC/d | Community production |
| MLD | m | Mixed layer depth |
| ben_DIC | mmol/m^2 | Benthic Carbon Dioxide |
| ben_N | mmol/m^2 | Benthic Nitrate - Aerobic Layer |
| ben_NH4 | mmol/m^2 | Benthic Ammonium - Aerobic Layer |
| ben_P | mmol/m^2 | Benthic Phosphate - Aerobic Layer |
| ben_Si | mmol/m^2 | Benthic Silicate - Aerobic Layer |
| ben_O | mmol/m^2 | Benthic Oxygen |
| ben_dep | mg/m^2 | Deposit Feeders, Macrobenthos C |
| ben_sus | mg/m^2 | Suspension / Filter Feeders, Macrobenthos C |
| ben_DOC | mg/m^2 | Dissolved Detrital Carbon |
| optChl | mgC/m^2 | Chlorophyll-a averaged over the optical depth |
| optDepth | m | Optical Depth (i.e. depth over which light reduces by factor e) |

Available at depth zones (m): 5, 30, 75, 150, 350, 750, 2000

**Figures**


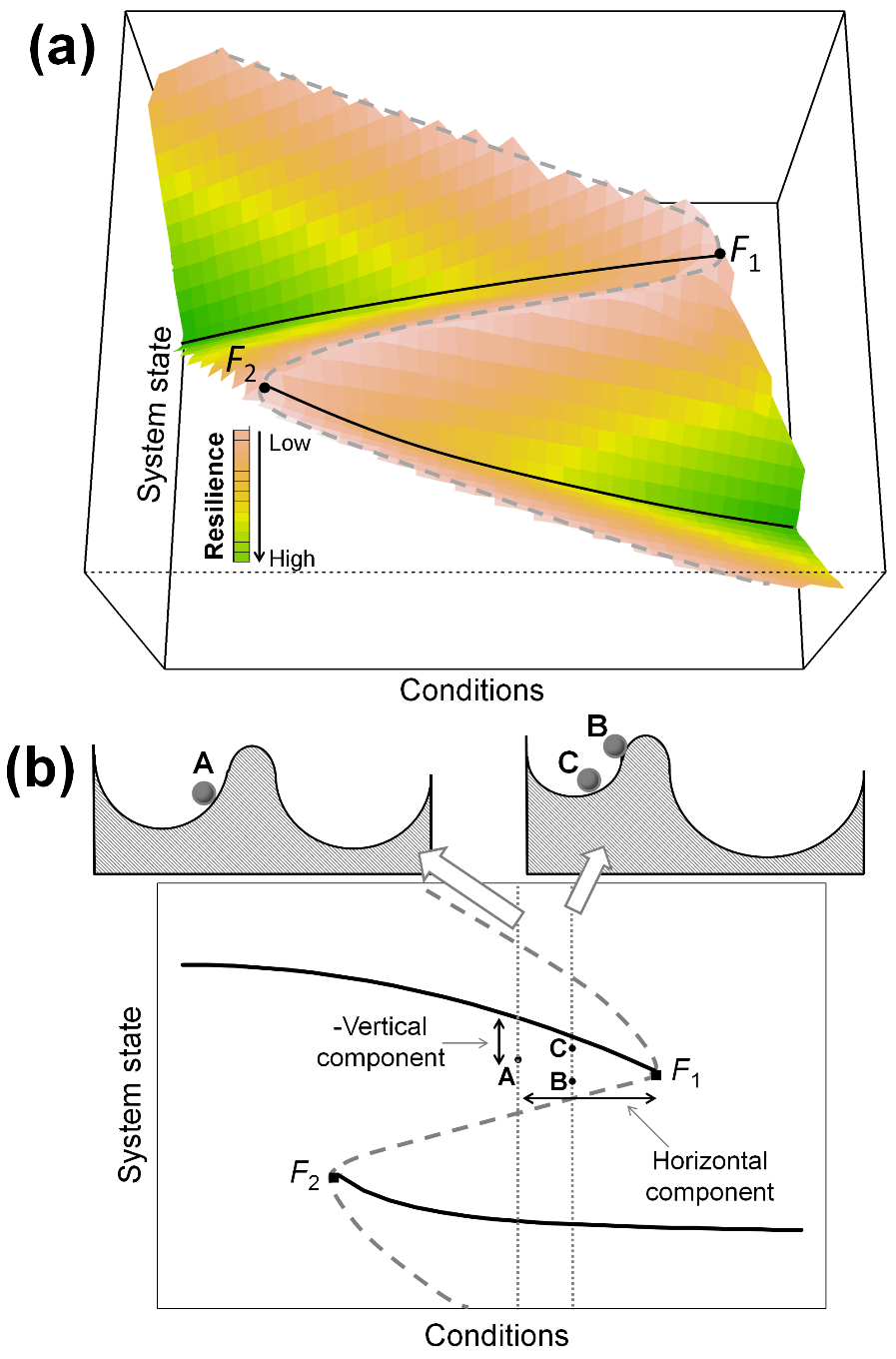


**Figure S0.** A generic folded stability landscape with two basins of attraction. System response curves (attractors) and basins’ borders are indicated by black continuous and grey dashed lines, respectively. As conditions (stressors) change and the system approaches a tipping point (F1 or F2) resilience erodes and the basins of attraction become narrower and shallower (a). In the context of the Integrated Resilience Assessment (IRA) framework, the horizontal distance of a state from the tipping point expresses its horizontal component of resilience (hComp), while the distance of a state from its attractor expresses its negative vertical component of resilience (-vComp) (b). The negative sign indicates that the greater the distance of a state from its attractor, the lower its resilience. Resilience of a state ‘A’ is then estimated as *Res*A = *hComp*A + *vComp*A. States ‘B’ and ‘C’ have the same hComp but different vComp; hence, state ‘C’ is more resilient than state ‘B’. States ‘A’ and ‘B’ have the same *vComp* but different *hComp*; hence, state ‘A’ is more resilient than state ‘B’. Adjusted from Vasilakopoulos & Marshall (2015).


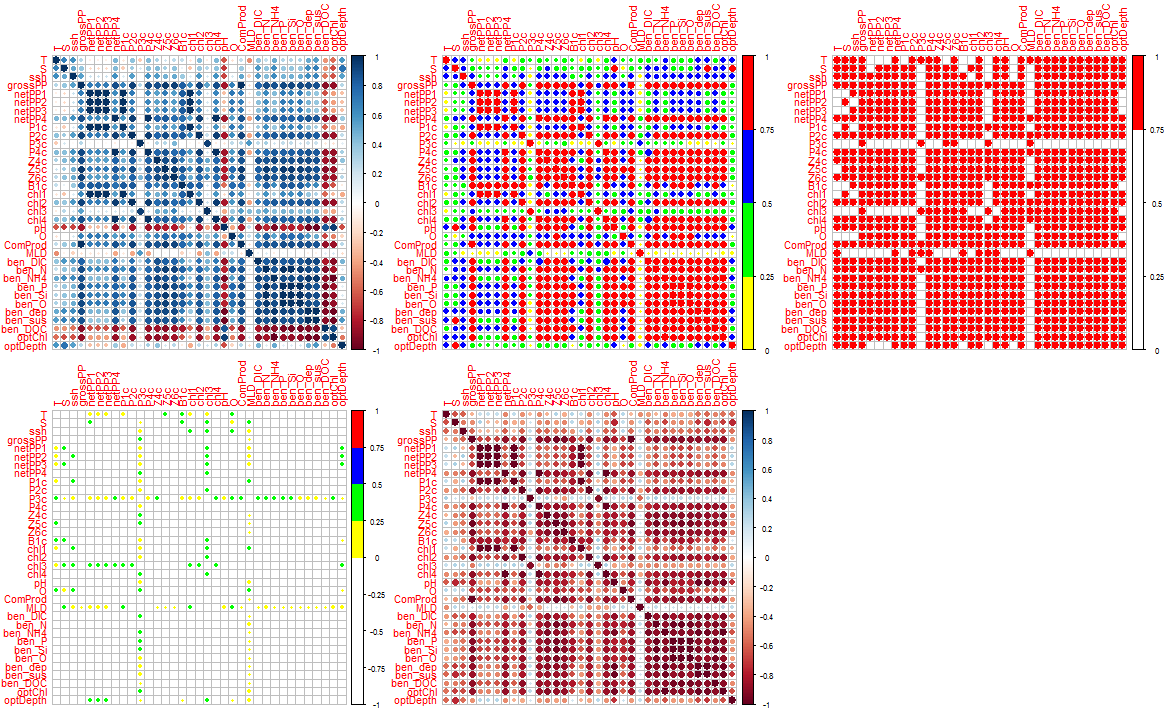


**Figure S1.** Correlation plots of the investigated environmental indices.

1. (top left) Simple correlation plot/Pearson (white points are not correlated)
2. (top mid) Distance correlation plot (yellow points are not correlated)
3. (top right) Significance test plot (white points is where we don't reject the null hypothesis -independence)
4. (bottom left) Distance correlation for *p* <= sig_level (Ignore white points- they reject null hypothesis of independence. Where do we have independence? yellow, green points
5. (bottom mid) Significance test plot (red is 'dependent', Blue and low value is independent and sig independent)

Note on Fig. S1

Since many of the environmental indices may record similar information, including multiple sources of similar information will not improve the model. It was therefore important to only include indices that contained information not included by other sources.

The relationships between the indices were explored. Of particular interest is the association between pairs of indices. Many methods are available to quantify the strength of association between a pair of variables. The most familiar method is correlation. However, correlation is only for quantifying the strength of a linear relationship. In the real world, most relationships are non-linear and are only approximately linear over a small range meaning that correlation must be used with care.

It must be remembered that correlation does not imply causation.

Here, each pair of environmental indices is analysed using ‘distance correlation’. Of particular interest is pairs of indices that appear to be independent (have low values of distance correlation) as these will each contain new information. The power of distance correlation is enough to avoid Type II errors for many relationship types.

However, it is possible that the distance correlation score between two independent data sets can be greater than 0, implying dependence i.e. the chance of a Type I error (a false positive).

The *dcov.test*() in R function provides a test of multivariate independence using a permutation bootstrap method. It returns a *p*-value that can be used for significance testing. The null hypothesis is that the data are independent. Performing this test will help avoid Type I errors i.e. avoid mistakenly rejecting the null (independence) hypothesis.


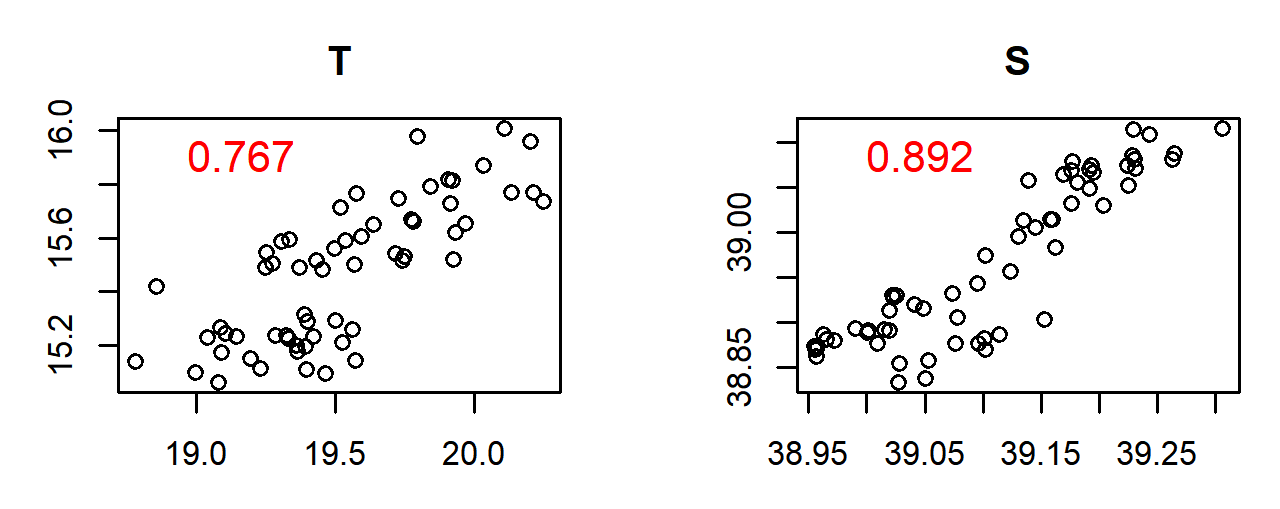


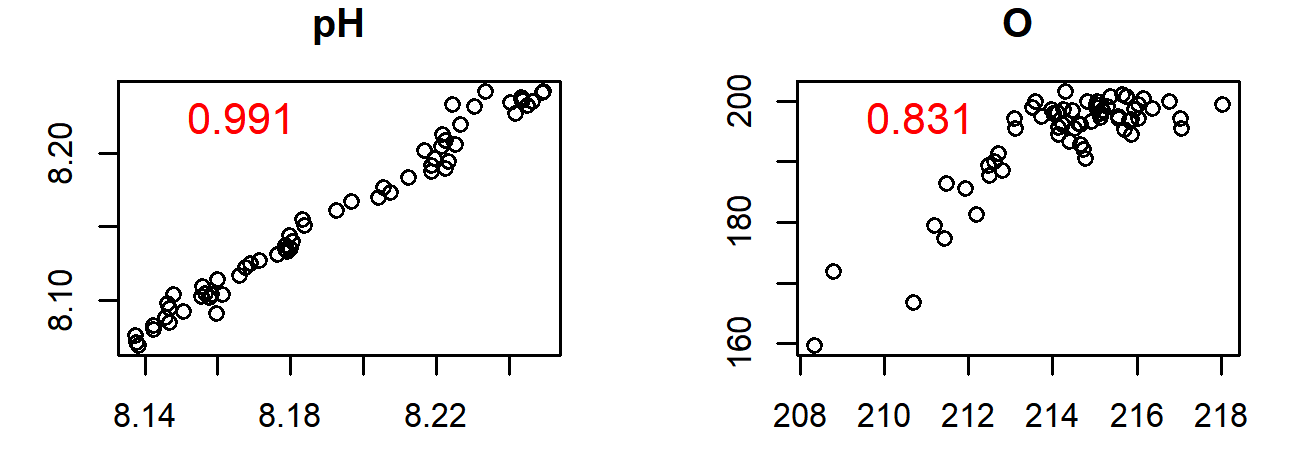


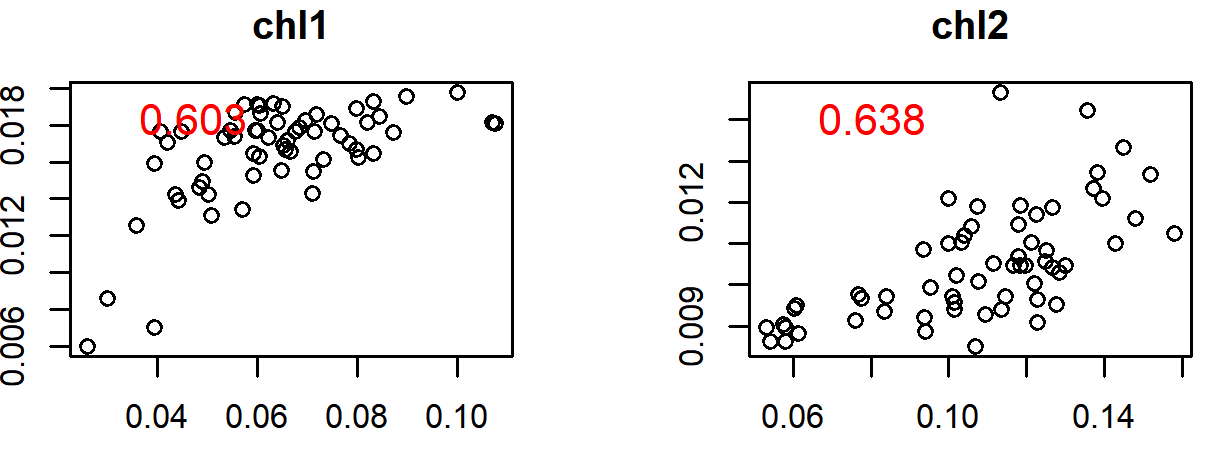


**Figure S2.** Correlation plots for selected environmental indices between two depth zones: surface values (5m) (x-axis) and values at the bottom depth (y-axis). Each dot represents a year from 1966 to 2017.

**Figure S3.** Correlation plots between

(top) LPUC (HELSTAT – kg/GT) and LPUE (EU DCF – kg/GTxDAS) for the Aegean Sea demersal resources during 2003 and 2016

(bottom) LPUC (HELSTAT – kg/GT) and LPUE (EU DCF – kg/kWxDAS) for the Aegean Sea demersal resources during 2009 and 2016


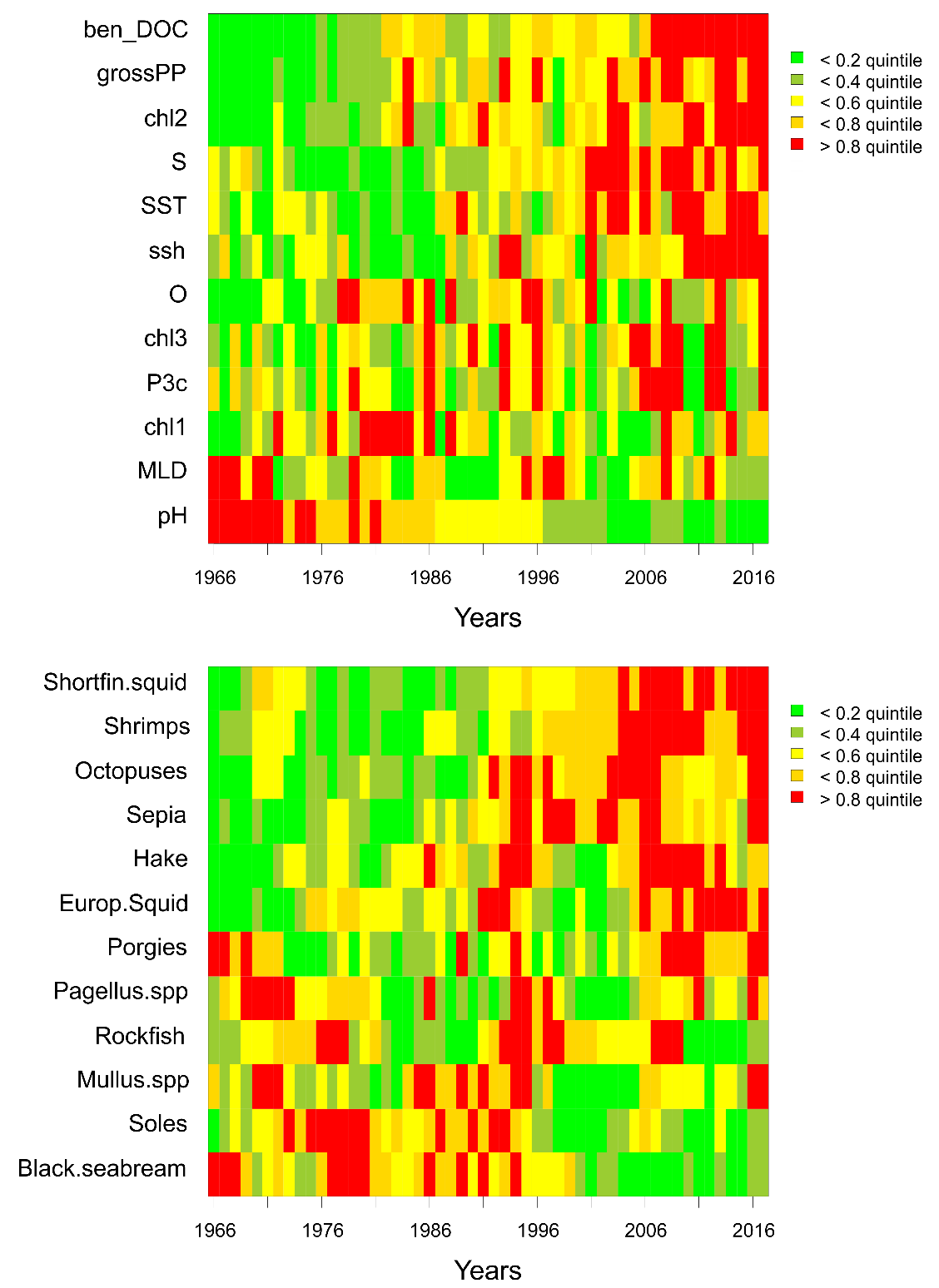


**Figure S4**. Traffic light plots for the 12 environmental indices (top) and the 12 demersal species/taxa LPUCs (bottom) from the Aegean Sea during 1966-2017. Temporal development of values sorted according to their loadings on the first principal components of PCAstr and PCAsys respectively.


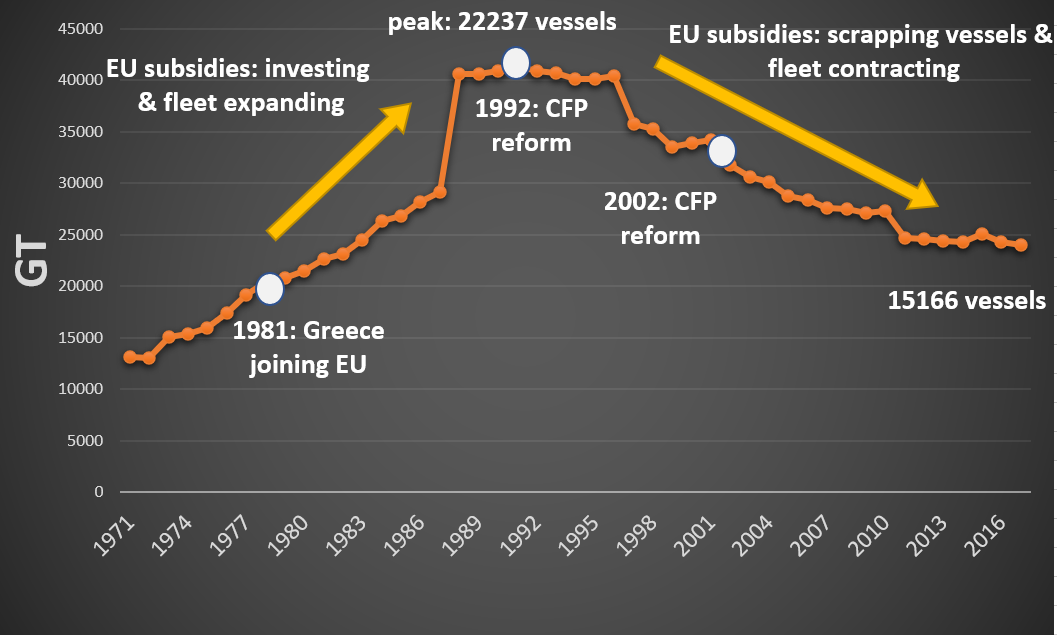


**Figure S5.** Evolution of Greek fleet in terms of capacity (GT)
